# The dehydration stress of couch grass is associated with its lipid metabolism, the induction of transporters and the re-programming of development coordinated by ABA

**DOI:** 10.1186/s12864-018-4700-3

**Published:** 2018-05-02

**Authors:** Anna Janská, Pavel Svoboda, Vojtěch Spiwok, Ladislav Kučera, Jaroslava Ovesná

**Affiliations:** 1 Faculty of Science, Charles University, Prague, Czech Republic; 2 Division of Crop Genetics and Breeding, Crop Research Institute, Prague, Czech Republic; 3 Factulty of Food and Biochemical Technology, University of Chemistry and Technology, Prague, Czech Republic

**Keywords:** Couch grass, Rhizome, Barley, Crown, Drought, Microarray, Dehydration stress

## Abstract

**Background:**

The wild relatives of crop species represent a potentially valuable source of novel genetic variation, particularly in the context of improving the crop’s level of tolerance to abiotic stress. The mechanistic basis of these tolerances remains largely unexplored. Here, the focus was to characterize the transcriptomic response of the nodes (meristematic tissue) of couch grass (a relative of barley) to dehydration stress, and to compare it to that of the barley crown formed by both a drought tolerant and a drought sensitive barley cultivar.

**Results:**

Many of the genes up-regulated in the nodes by the stress were homologs of genes known to be mediated by abscisic acid during the response to drought, or were linked to either development or lipid metabolism. Transporters also featured prominently, as did genes acting on root architecture. The resilience of the couch grass node arise from both their capacity to develop an altered, more effective root architecture, but also from their formation of a lipid barrier on their outer surface and their ability to modify both their lipid metabolism and transporter activity when challenged by dehydration stress.

**Conclusions:**

Our analysis revealed the nature of dehydration stress response in couch grass. We suggested the tolerance is associated with lipid metabolism, the induction of transporters and the re-programming of development coordinated by ABA. We also proved the applicability of barley microarray for couch grass stress-response analysis.

**Electronic supplementary material:**

The online version of this article (10.1186/s12864-018-4700-3) contains supplementary material, which is available to authorized users.

## Background

Drought stress represents the commonest agent of abiotic stress in plants. As a consequence of the changing climate, it is likely to become an even more regular feature in regions, which currently experience it only occasionally [1–3]. The implication is that crop improvement programs will need to increasingly prioritize drought tolerance as a breeding goal, while at the same time retaining the potential to yield well in the absence of the stress. While it may be possible to achieve these breeding goals empirically, more rapid progress should be possible if the mechanistic basis of drought tolerance were better understood. Much research effort continues to be expended in this direction in the major crop species, as well as in their model species. However, despite the recognition that many crop wild relatives are more resilient than the crop species themselves, little attention has been paid to exploring tolerance mechanisms in these species. A particular example is the small grain cereal relative couch grass (CG) (*Elymus repens*, syn. *Elytrigium repens*, *Agropyron repens* and *Triticum repens*). This perennial, hexaploid Triticeae species (the same tribe to which the three leading small-grained temperate cereals wheat, barley, wheat and rye belong) has been ranked among the three most serious weed species, infesting 37 crops across 65 countries [4]. Its strong competitive ability derives at least in part from its formation of rhizomes, which are highly tolerant of prolonged periods of moisture stress, and readily regenerate into whole plants when moisture becomes available.

The transcriptomic response of a number of plant species to drought or dehydration stress has been explored in depth in recent years, thanks to the development of genomic tools such as the DNA microarray. As one of the three sub-genomes of CG (H) is closely related to the barley genome [5–7], the assumption is that tools developed for barley should be informative in CG. The success of this heterologous approach has already been demonstrated in the genus *Sorghum,* where microarrays developed for *S. bicolor* have been used to investigate the transcriptome of its perennial, rhizomatous wild relative *S. propinquum* [8]. Similarly, the *Arabidopsis thaliana* microarray has been exploited to carry out transcriptomic analyses of a range of close and even rather distant relatives [9–11], while a soybean micro-array has been shown to be functional in common bean [12] and a tomato microarray in potato [13], pepper and eggplant [14] and strawberry [15].

Here we tested the hypothesis that the dehydration stress response of barley crown and the node of its wild extremely tolerant relative (couch grass) is different. We discuss mechanisms of acclimation to dehydration which play a crucial role in couch grass node, plant part critical for whole plant survival, similar to barley crown. We suggest the critical role of lipid metabolism, the induction of transporters and the re-programming of development coordinated by ABA (abscisic acid) in the dehydration stress response of couch grass leading to drought stress tolerance. These results could be interesting for barley breeding programs to develop drought tolerant genotypes, enable novel insight into adaptation of extremely tolerant plant species and demonstrate the applicability of barley microarray for couch grass transcriptome analysis. The dehydration stress responses of two barley cultivars contrasting with respect to drought tolerance have been described in our previous study [16].

## Results

### Relative water content, electrolyte leakage and re-sprouting

**Fig. 1:**
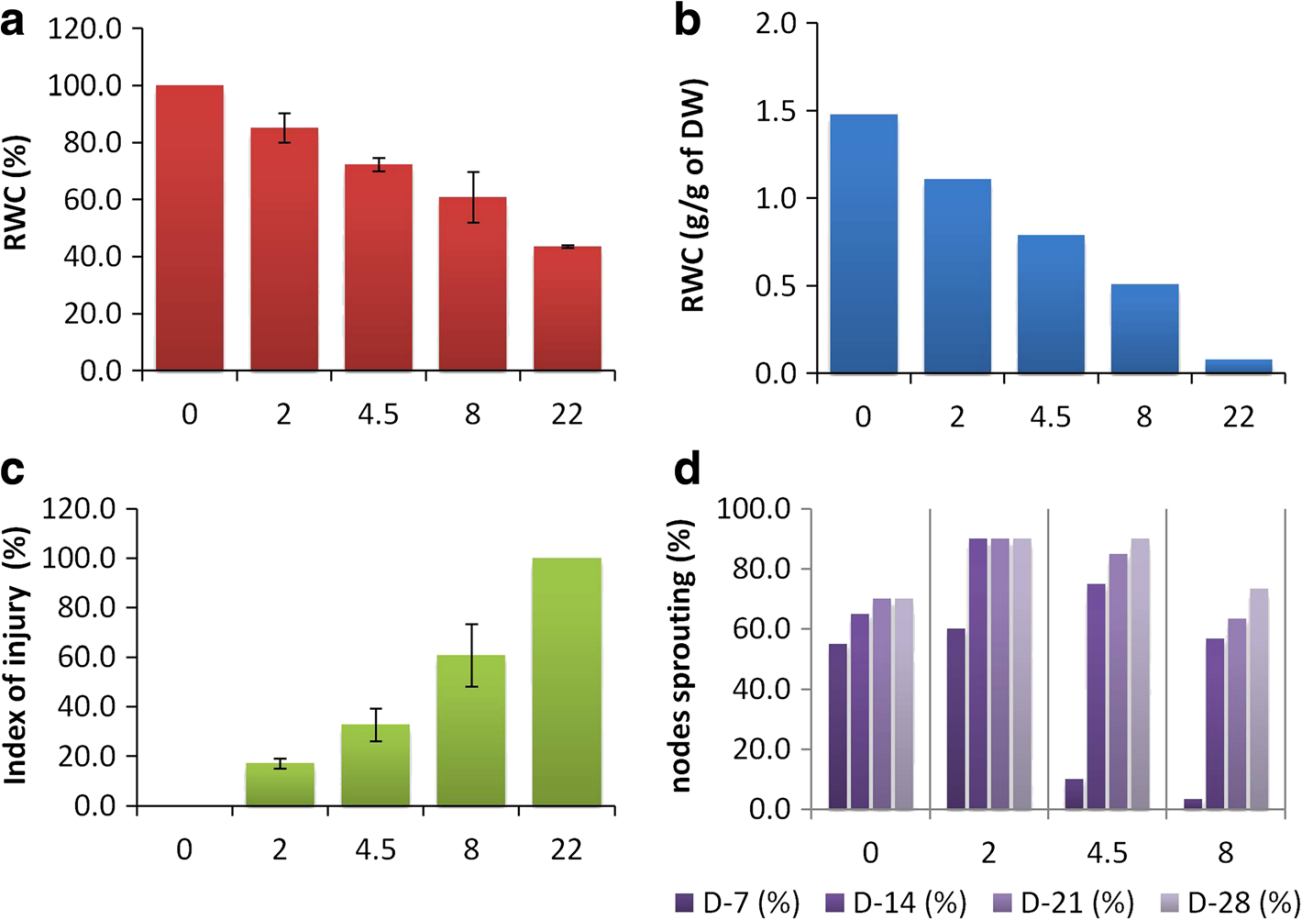
The physiological status of the dehydration-stressed rhizomes. **a**, **b** Water contents expressed as (**a**) a relative water content in % and (**b**) g per g DM. **c** Electrolyte leakage expressed in the form of an injury index. **d** The proportion of rhizomes able to regenerate new shoots when re-watered. The *x* axis depicts the duration of stress in h; D-7,D-14,D-22, D-28 represents the number of days after replanting

Before the stress was applied, the CG rhizome segments had an relative water content (RWC) of 59.6% (Fig. 1a), equivalent to a water content of 1.48 g per g dry matter (DM) (Fig. 1b). After exposure to the mild dehydration treatment (2 h at 28 °C), the RWC declined to 44.7% (1.11 g per g DM); the medium treatment (4.5 h) further decreased RWC to 31.8% (0.79 g per g DM), and the severe treatment (8 h) to 20.5% (0.51 g per g DM). After 22 h, the RWC reached 3.2% (0.08 g per g DM). After the latter treatment, the rhizome segments were no longer viable. The response of *I*_*t*_ to the treatments is shown in Fig. 1c. The range was from 0% (non-treated control) to 100% (dead). After the mild stress, this index rose to 17.2%, after the medium stress to 32.8% and after the severe stress to 60.9%. When the rhizome segments were replanted, by seven days, 55% of the non-treated ones had sprouted, rising to 70% after 21 days; the proportion did not rise further (Fig. 1d). For the treated segments, the proportions which re-sprouted after seven days were 60% (mild), 10% (medium) and 3% (severe). The effect of stress was of short duration, as by 28 days, 90% of the segments exposed to the mild and medium stress levels were able to re-sprout, while the re-sprouting proportion of the severely stressed segments was 73%.

### Global comparison of transcription profiles and data quality

**Fig. 2:**
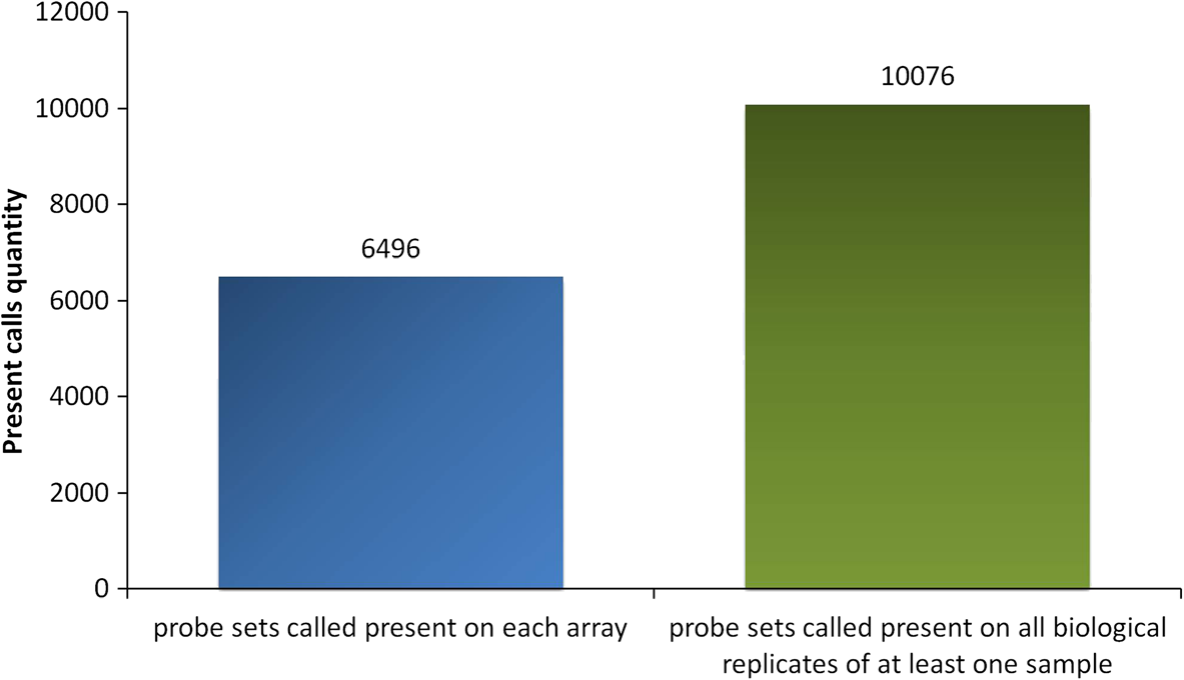
Statistics relating to “present calls” when CG transcripts were hybridized to the barley microarray. “Present calls” corresponds to probe sets outputting a signal intensity significantly higher for perfect matches (100% sequence complementarity to the reference sequence) than for mismatches (MM - differ from the perfect match probes by a single base substitution at the centre base position, disturbing the binding of the target gene transcript. MM serve as a control for cross-hybridization). The blue bar represents the numbers of probe sets called as present on each array, while the green bar displays the number of probe sets called as present on all biological replicates of at least one sample. The numbers on the y axis depict the quantity of probe sets

Microarray raw data analysis using MAS 5.0 algorithm revealed that nearly half (10076) of all features on barley1 GeneChip (22840) were called as present on all biological replicates of at least one sample once hybridized with CG RNA (Fig. 2). In addition, majority of those features (6496) were called present on all arrays (Fig. 2). These probe sets were considered to detect barley homologs within the CG genome involved in stress response and confirmed the applicability of barley1 GeneChip for the CG stress response analysis.

### The identification of differentially transcribed genes (DTGs)

**Fig. 3:**
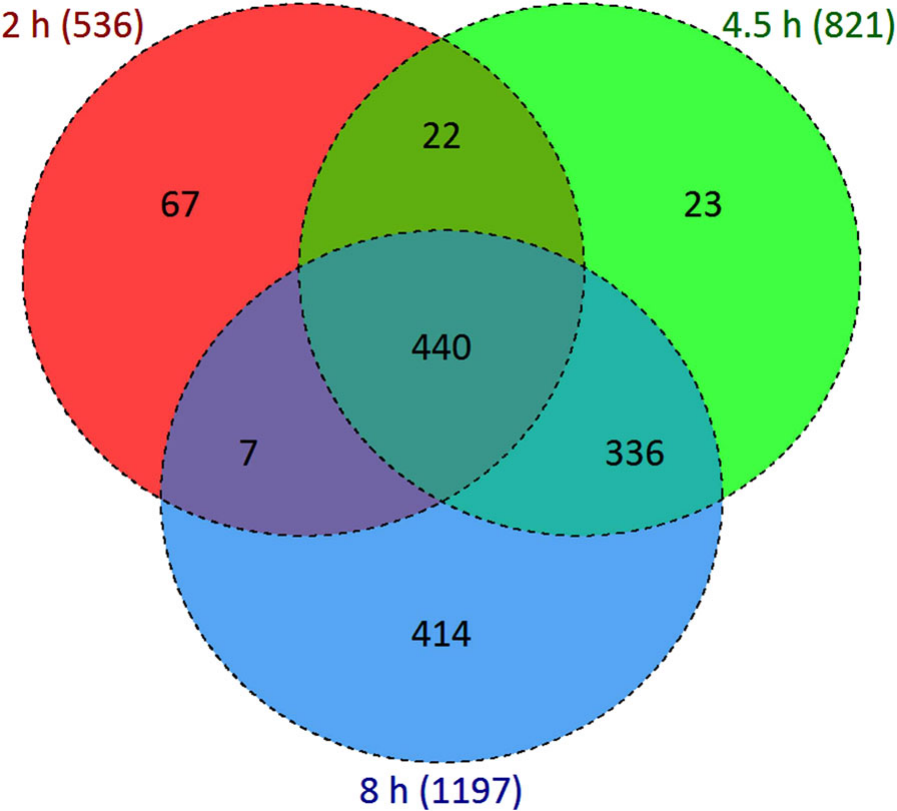
The specificity and commonality of DTGs in the dehydration treated CG rhizomes

**Fig. 4:**
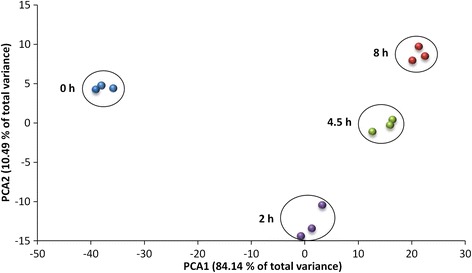
PCA applied to the set of all DTGs. The *x* and *y* axes correspond to the two main components. The data in parentheses indicate the contribution of the given component to the overall variance. Circles represent individual samples, while points within each circle represent the biological replicates. Blue points on the left side correspond to non-treated samples, while the purple, green and red points on the right side represent the three simulated dehydration treatments

Through use of the LIMMA algorithm, 1309 probe sets were identified as being significantly altered between treated and non-treated samples (Fig. 3). Of these, 536 (40.9%) related to the contrast non-treated vs mildly stressed rhizome segments, 821 (62.7%) to the contrast with the medium stress level and 1197 (91.4%) with the severe stress level. About 40% of the DTGs were identified at only a single stress level (67 at the mild level, 23 at the medium level and 414 at the severe level), while ~ 34% were detect at all three levels, and the remaining were detected at two of the three levels (22 shared by the mild and medium levels, 336 by the medium and severe levels and 7 to the mild and severe levels). Of the 440 DTGs associated with all three stress levels, 283 were up-regulated by the stress and 157 down-regulated. Among the former, 162 showed a progressive enhancement in transcription as the stress level was increased (Table 1). The PCA (principal component analysis) applied to the set of DTGs is illustrated in Fig. 4. The first two components explained, respectively, 84.0% and 10.5% of the total variance. Variation between biological replicates represented only a small proportion of the total, confirming the datasets quality. There were significant differences not only between the non-treated and treated rhizome segments, but also between treatments.

**Table 1:** Selected DTGs whose transcription was enhanced by increasing the severity of the dehydration treatment

ID^a^	Log2FC^b^			Affymetrix annotation^d^	AGI^e^
	2^c^	4.5^c^	8^c^		
HT11N18r_s_at	5.19	5.60	5.77	9-cis-epoxycarotenoid dioxygenase	AT1G78390.1
Contig1718_s_at	4.17	5.16	5.18	dehydrin 9	AT3G50980.1
Contig2406_at	3.75	4.88	5.16	ABA-inducible protein WRAB1	AT3G15670.1
Contig7672_at	3.92	4.78	4.85	protein phosphatase 2C	AT4G31750.1
Contig8184_at	3.97	4.50	4.50	WSI18 protein	AT3G15670.1
HV06O23u_at	3.60	3.95	3.98	mRNA cleavage factor subunit	AT4G29820.1
Contig13161_at	2.88	3.73	3.94	protein phosphatase 2C-like protein	AT1G72770.3
Contig1713_s_at	2.78	3.75	3.80	dehydrin 4	AT5G66400.1
Contig14720_s_at	1.87	3.14	3.74	putative phosphoinositide kinase	AT1G34260.1
HV10J12u_s_at	2.70	3.45	3.71	actin depolymerization factor-like	AT5G59880.1
Contig14870_at	3.46	3.65	3.71	putative trehalose-6-phosphate	AT4G12430.1
Contig2407_s_at	2.42	3.43	3.70	ABA-inducible protein PHV A1	AT3G15670.1
Contig1708_s_at	2.07	3.37	3.60	dehydrin 6	AT4G01985.1
Contig1701_s_at	1.97	3.10	3.47	dehydrin 2	AT3G50980.1
Contig1724_s_at	2.66	3.11	3.40	dehydrin 3	AT5G66400.1
Contig2405_at	2.33	3.22	3.34	group 3 LEA protein	AT3G15670.1
Contig10934_at	2.56	3.09	3.32	Putative abscisic acid-induced protein	AT3G22490.1
Contig8905_at	1.56	3.02	3.31	xylanase inhibitor protein I	
rbaal10h14_at	1.07	2.27	3.30	abscisic acid-induced protein	
Contig3807_at	1.78	2.85	3.26	putative nifU-like protein	AT4G22220.1
Contig8220_at	2.49	2.97	3.17	late embryogenesis abundant protein	AT4G21510.1
HVSMEm0008B04r2_s_at	2.28	3.06	3.14	UDP-glucose 4-epimerase	AT4G23920.1
Contig5724_at	1.62	2.50	2.96	4-hydroxyphenylpyruvate dioxygenase	AT1G06570.1
Contig3810_at	1.75	2.89	2.93	WSI76 protein/Galactinol synthase	AT1G09350.1
Contig4942_at	1.46	2.46	2.89	ATP-dependent Clp protease	AT5G51070.1
Contig4955_s_at	2.44	2.82	2.87	putative sugar-starvation induced	AT2G32150.1
Contig26196_at	1.89	2.75	2.86	putative raffinose synthase	
EBro08_SQ007_B12_s_at	1.32	2.02	2.77	ABA-inducible protein WRAB1	AT3G15670.1
Contig6110_at	1.15	2.36	2.67	Ca2 + −dependent lipid-binding protein	
Contig4760_s_at	2.10	2.62	2.63	putative late embryogenesis abundant	AT1G01470.1
Contig2924_s_at	1.18	2.28	2.61	aldehyde dehydrogenase homolog Dha1	AT1G54100.2
Contig10022_at	1.33	2.11	2.61	putative glycine-rich cell wall protein	AT5G53870.1
HF18A22r_s_at	1.54	2.34	2.61	Formate dehydrogenase, mitochondrial	AT5G14780.1

^a^Affymetrix 22 K Barley1 GeneChip Genome Array probe set ID

^b^Log_2_ transformed fold change of treated samples against controls

^c^duration of stress (h)

^d^Microarray manufacturer’s annotation of individual IDs

^e^*A. thaliana* locus identifier corresponding to individual IDs

### Functional analysis of the set of DTGs

**Fig. 5:**
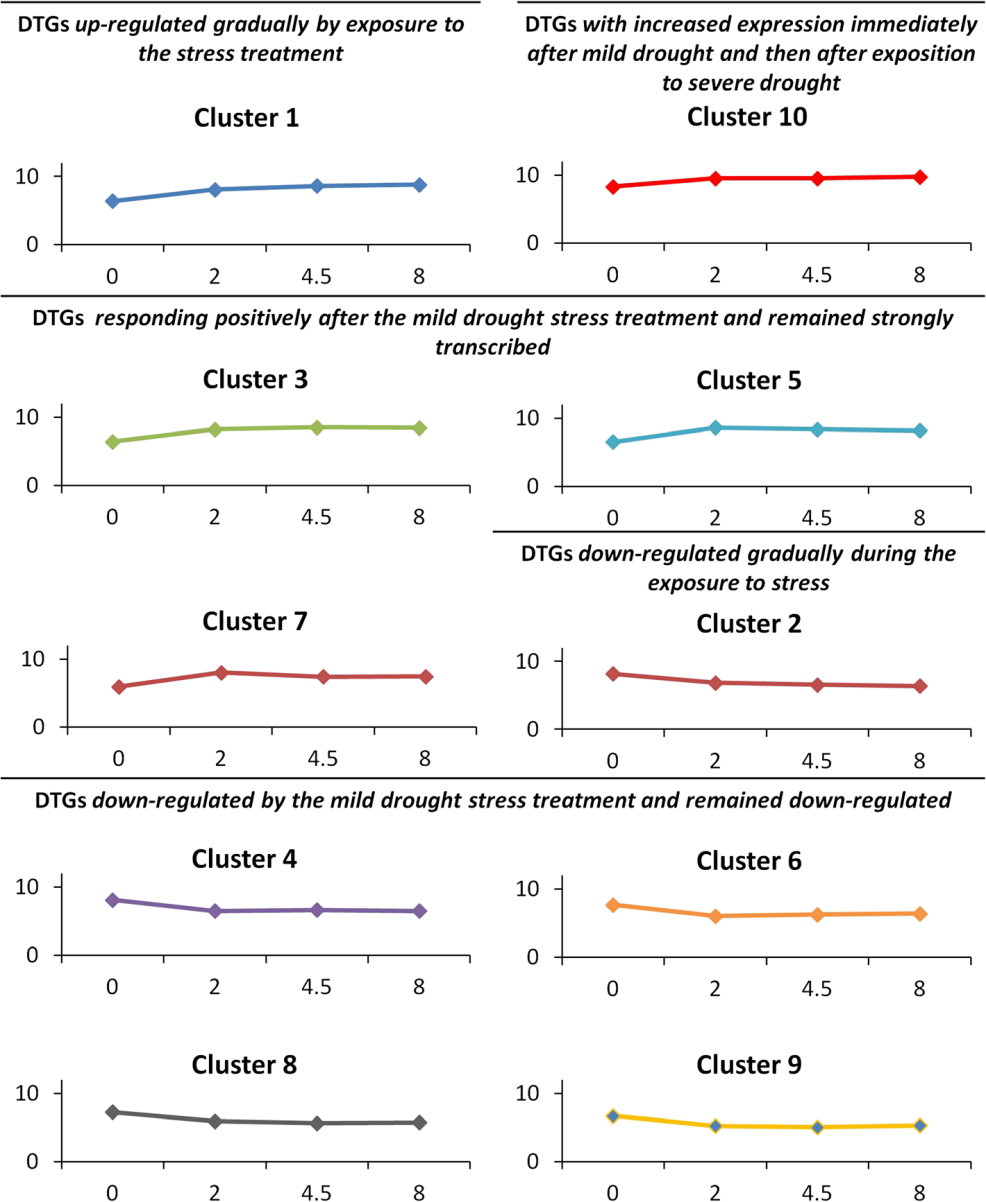
A cluster analysis of the DTGs common to all tree treatments. The charts display the mean transcript abundance (*y* axis) for genes grouped into a given cluster

The DTGs associated with all three stress levels were grouped into ten clusters on the basis of their response to an increasing level of stress (Fig. 5; Additional file 1: Table S3, Additional file 2: Table S4, Additional file 3: Table S5, Additional file 4: Table S6, Additional file 5: Table S7, Additional file 6: Table S8, Additional file 7: Table S9, Additional file 8: Table S10, Additional file 9: Table S11, and Additional file 10: Table S12).

Cluster #1 (162 DTGs; Table 1; Additional file 1: Table S3) was formed by genes up-regulated gradually by exposure to the stress treatment, and whose products are likely relevant for either the response to osmotic stress and/or are promoted by abscisic acid (ABA). About a quarter of the products of these genes (43 DTGs) could be associated with catalytic activity: for example, 13 are oxidoreductases, and 42 are expressed in membrane-bounded organelles. One of the most strongly stimulated genes encodes 9-*cis*-epoxycarotenoid dioxygenase (NCED; AT1G78390.1), an enzyme which catalyzes the first step of ABA synthesis; other stress up-regulated ABA-inducible genes included several encoding so-called “late embryogenesis abundant” (LEA) proteins which, under conditions of osmotic stress, inhibit the loss of activity of a number of enzymes. Examples of these are several members encoding Y_n_Sk_m_ dehydrins (Table 2), documented to accumulate in barley plants in response to dehydration [17]. The genes encoding HVA1 (AT3G15670.1), WSI18 (AT3G15670.1) and the LEA14-A protein (AT1G01470.1) were also up-regulated by the stress. Several of the Cluster #1 DTGs encoded proteins/enzymes involved in carbohydrate metabolism, namely UDP-glucose 4-epimerase (AT4G23920.1*)*, a putative trehalose-6-phosphate phosphatase (AT4G12430.1), galactinol synthase (AT1G09350.1*)* and a putative raffinose synthase (AT5G40390.1). A gene encoding pyrroline-5-carboxylate synthetase (AT2G39800.1*)* also belonged to this cluster, as did genes encoding proteins involved in stress signal transduction such as protein phosphatase 2C (AT1G72770.3, AT4G28400.1, AT4G28400.1, AT4G31750.1, AT1G72770.3), phosphatidylinositol-3-phosphate 5-kinase (PI3PK; AT1G34260.1), MAP kinase (AT1G10210.2), Ser/Thr protein kinase (AT5G18610.1, AT5G56460.1) and a putative calcium-binding protein (AT4G26470.1, AT4G38810.2).

**Table 2:** DTGs encoding dehydrins

ID^a^	Log2 FC^b^			Affymetrix annotation^d^	Structural type^e^	AGI^f^
	2^c^Hrs	4.5^c^	8^c^			
Contig1725_s_at	0.162	0.920	1.067	DHN1	*Y*_*n*_ *SK*_*m*_	AT5G66400.1
Contig1721_at	0.916	1.752	2.090	DHN2	*Y*_*n*_ *SK*_*m*_	AT5G66400.1
Contig1701_s_at	1.972	3.100	3.472	DHN2	*Y*_*n*_ *SK*_*m*_	AT3G50980.1
Contig1724_s_at	2.662	3.115	3.402	DHN3	*Y*_*n*_ *SK*_*m*_	AT5G66400.1
Contig1713_s_at	2.785	3.751	3.810	DHN4	*Y*_*n*_ *SK*_*m*_	AT5G66400.1
Contig1717_s_at	0.893	1.446	1.436	DHN5	*K*_*n*_	AT3G50970.1
Contig1708_s_at	2.077	3.379	3.603	DHN6	*Y*_*n*_ *SK*_*m*_	AT4G01985.1
Contig1709_at	1.603	2.141	2.300	DHN7	*Y*_*n*_ *SK*_*m*_	AT5G66400.1
Contig2855_at	1.095	1.199	0.987	DHN8	*SK*_*n*_	AT1G20440.1
Contig1718_s_at	4.174	5.168	5.185	DHN9	*Y*_*n*_ *SK*_*m*_	AT3G50980.1
Dhn10(Morex)_s_at	1.262	1.846	2.090	DHN10	*Y*_*n*_ *SK*_*m*_	AT3G50970.1
Contig10207_s_at	0.687	1.089	0.978	DHN11	*Y*_*n*_ *SK*_*m*_	AT5G66400.1

^a^Affymetrix 22 K Barley1 GeneChip Genome Array probe set ID

^b^Log_2_ transformed fold change of treated samples against controls

^c^duration of stress (h)

^d^Microarray manufacturer’s annotation of individual IDs of individual dehydrins (DHNs)

^e^Structural type of individual dehydrins

^f^*A. thaliana* locus identifier corresponding to individual IDs

Clusters #3 (81 DTGs; Additional file 3: Table S5), #5 (35 DTGs; Additional file 5: Table S7) and #7 (three DTGs; Additional file 7: Table S9) included genes responding positively after the mild dehydration stress treatment and remained either strongly transcribed, or - as those grouped into Cluster #5 – showed a peak level of transcription in the medium dehydration stress treatment. Products of these genes are involved in signaling (seven DTGs) or the response to ABA treatment (six DTGs) and/or the imposition of osmotic (five DTGs) or low temperature (six DTGs) stress; five of these genes are known to encode transporters. The two genes grouped into Cluster #10 (Additional file 10: Table S12) were both rapidly up-regulated by moisture stress. One of the two genes is a typical “stress response” gene, encoding an LTI (or Blt 101.1, low temperature induced) protein (AT2G38905.1) and the other encoded an unknown protein (HS07G10u_s_at).

Cluster #2 (86 DTGs; Additional file 2: Table S4) groups genes down-regulated gradually during the exposure to stress: their products are associated with carbohydrate metabolism (ten DTGs), alcohol catabolism (six DTGs), catalysis (32 DTGs) and transferase (14 DTGs). The products of 20 of these genes are thought to localize to the cytoplasm.

Clusters #4 (24 DTGs; Additional file 4: Table S6), #6 (18 DTGs; Additional file 6: Table S8), #8 (24 DTGs; Additional file 8: Table S10) and #9 (five DTGs; Additional file 9: Table S11) harbor genes, which were down-regulated by the mild dehydration stress treatment and remained down-regulated. A number of these genes encoded either proteins localized within an organelle or which possessed catalytic activity.

In all, there were 67 DTGs exclusively induced by the mild dehydration stress treatment (half up- and half down-regulated). Those which were up-regulated encoded products associated with cross membrane transport, specifically of citrate (putative transmembrane protein AT1G02260.1), amino acids and water (NOD26-like intrinsic protein 1;2, AT4G18910.1; δ tonoplast intrinsic protein, AT3G16240.1; plasma membrane intrinsic protein 2A (PIP2A), AT3G53420.2; amino acid transporter family protein, AT3G56200.1). Others encoded products, some of which localized to the cell wall, including extensin (AT4G13340.1), expansin (AT1G69530.2) and glycosyl hydrolase (AT1G78060.1); others were implicated in the abiotic stress response (ascorbate peroxidase (AT1G07890.8)) and still others in ABA signaling (ABRE binding factor 4 (AT3G19290.1); protein phosphatase 2CA (AT3G11410.1)). The group of down-regulated genes included three encoding small heat shock proteins (AT5G59720.1; AT4G27670.1; AT3G46230.1) and one a fatty acid desaturase (AT5G05580.1). Of the 23 genes exclusively induced by the medium stress treatment, the up-regulated group included genes encoding dehydrin (AT5G66400.1), chitinase (AT2G43590.1), glutamine-dependent asparagine synthase 1 (AT3G47340.1) and oxidative stress 3 (AT5G56550.1), while the down-regulated group included genes encoding 3-ketoacyl-CoA synthase 20 (AT5G43760.1) and peroxidase 21 (AT2G37130.2). A much larger group was formed by the 414 genes exclusively induced by the severe dehydration stress treatment. Of these, 189 were up- and 225 down-regulated. Many (53) of the up-regulated group encoded proteins having catalytic activity, nine are involved in active transmembrane transport, 44 localize to the cytoplasm, 43 are expressed within an organelle and seven within the vacuole. Some of these genes encode enzymes involved in the synthesis of phenolic compounds, such as cinnamyl alcohol dehydrogenase (AT4G37980.1), arogenate dehydratase 4 (AT3G44720.1), fumarylacetoacetate hydrolase-like protein (AT1G12050.1) and cinnamate-4-hydroxylase (AT2G30490.1), and some in lipid metabolism, e.g. phospholipase D (AT3G16785.1), esterase (AT5G41120.1), long-chain-fatty acid CoA ligase family protein (AT2G04350.2). Among the down-regulated group, there was an over-representation of genes encoding products associated with anatomical structure development (19 DTGs), transport (17 DTGs, of which 13 have substrate-specific transporter activity) and nitrogen metabolism (29 DTGs). There were also a number of genes the products of which participate in translation (19 DTGs), especially the structural constituents of the ribosome (16 DTGs).

### The comparative response of CG and barley

**Fig. 6:**
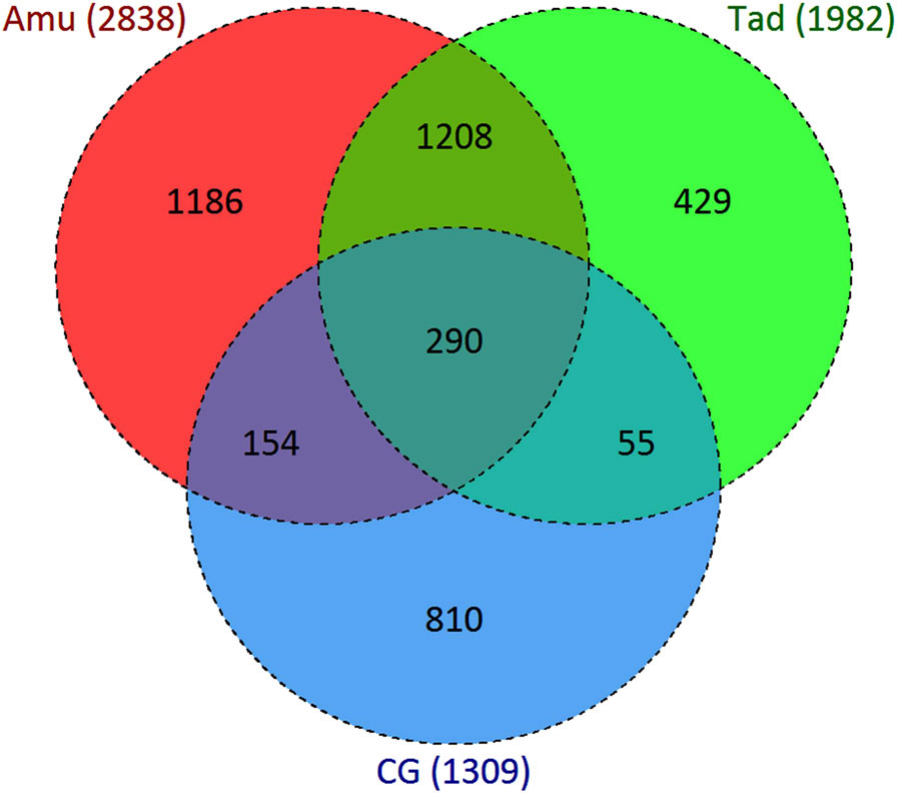
The specificity and commonality of DTGs in the dehydration treated rhizomes of CG and the crowns of the barley cultivars Tadmor and Amulet

The transcriptomic dehydration response of the crown of the two contrasting (with respect to drought tolerance) barley cultivars cv. Amulet (drought sensitive) and cv. Tadmor (drought tolerant) has been documented in our previous study [16]. Among the 4132 DTGs identified in barley, 290 were shared between CG and both barley cultivars, 55 between CG and cv. Tadmor, and 154 between CG and cv. Amulet. There were 810 DTGs, which were specific to CG (Fig. 6; Additional file 11: Table S13). Among the 290 common DTGs, there were 21 genes encoding transporters or proteins associated with transport and localization: of these, six are lipid transfer proteins. A second group of over-represented genes encoding proteins is involved in ABA signaling (12 DTGs), the response to moisture deficiency (ten DTGs) or oxidative stress (seven DTGs): most of these were up-regulated by the stress. Another shared up-regulated gene encoded spermidine synthase 3 (AT5G53120.5), an enzyme involved in polyamine synthesis. Some of the DTGs in this category were down-regulated in barley, but up-regulated in CG or vice versa; examples are genes encoding the transporters PIP aquaporin (PIP2A; AT3G53420.2), vacuolar iron transporter 1.1 (VIT1.1; AT2G01770.1) and a tonoplast intrinsic protein 1;3 (TIP1;3; AT4G01470.1), all of which were down-regulated in both barley cultivars, but up-regulated in CG in each of the three drought treatments. A similar behavior was exhibited by a gene encoding a dehydrin (AT1G54410.1), while the opposite behavior (up-regulated in both barley cultivars, but down-regulated in CG) was exhibited by a gene encoding an 18 KDa heat shock protein (AT5G59720.1).

The crown of the drought tolerant cv. Tadmor and the node of CG share a similar response to dehydration stress. In both, the set of over-represented DTGs involved those encoding products concerned with post-embryonic development (six genes), anatomical structure development (eight genes) and reproductive structure development (five genes). Among the genes down-regulated in both barley and CG were a gene encoding protein-arginine-N-methyltransferase (PRMT10; AT1G04870.2), associated with the vegetative to reproductive transition; a gene encoding transcriptional factor B3 family protein (NPH4; AT5G20730.2), involved in leaf development and lateral root primordium development; and a homolog of an *A. thaliana* gene encoding KNOTTED-LIKE HOMEOBOX (AT1G62990.1), involved in xylem development. Among those up-regulated in both cv. Tadmore and CG were a gene encoding oleosin1 (AT4G25140.1), responsible for lipid accumulation. Two genes showed contrasting behavior between cv. Tadmor and CG: these encoded jasmonate-zim domain protein1 (AT1G19180.2), involved in the jasmonate signaling pathway, which was down-regulated in cv. Tadmor, but up-regulated in CG; and a gene encoding late elongated hypocotyl (AT1G01060.4), a transcription factor responsive to a broad spectrum of phytohormones and salinity stress [18], which was up-regulated in cv. Tadmor and down-regulated in CG.

The most abundant group of CG-specific DTGs included those encoding products involved in carbohydrate metabolism (21 genes), nitrogen metabolism (25 genes) and lipid metabolism (26 genes). There was a particularly pronounced up-regulation of a gene encoding chloroplast β-amylase (AT4G17090.1), 1-deoxy-D-xylulose 5-phosphate reductoisomerase (AT5G62790.1) or glucosyl transferase family 8 (AT1670090.2), while genes encoding phosphofructokinases (AT4G26270.1; AT1G76550.1) were strongly down-regulated. Some genes encoding proteins involved in amino acid metabolism were also up-regulated, such as ornithine-delta-aminotransferase (AT5G46180.1) or protein named oxidative stress 3 (AT5656550.1). The significance of lipid metabolism in the drought acclimation process was highlighted by the up-regulation of genes encoding a phosphatidyl serine synthase (AT1G15110.1), a possible phosphatidylinositol-3P 5-kinase (or “forms aploid and binucleate cells 1A”; AT1G34260.1), 1-deoxy-D-xylulose 5-phosphate reductoisomerase (AT5G62790.1), NCED (AT1G78390.1) or squalene synthase (AT4G34640.1). Molecular function of the last one is unknown, but probably is involved in endomembrane homeostasis [19] Other over-represented groups of genes included those encoding proteins involved in the response to osmotic stress (35 genes), in the response to ABA (20 genes) and stomatal movement (six genes) as well as with localization (57 genes), developmental processes (62 genes) and signal transduction (eight genes). The importance of ABA in the drought stress responses was confirmed not only by the up-regulation of a number of ABA-dependent genes but also by the strong up-regulation of genes involved in ABA synthesis, such as those encoding NCED (AT1G78390.1) and zeaxanthin epoxidase (AT5G67030.1). On the other hand, most of the DTGs encoding products involved in developmental processes were down-regulated: examples were the genes encoding homeobox protein 16 (HB16; AT4G40060.1), RING IV-box superfamily protein (AT3G63530.2) and homeodomain-like superfamily protein (AT1G01060.4); the gene encoding choline/ethanolamine kinase 4 (CEK4; AT2G26830.1) was, however, strongly up-regulated. Similar down-regulation was observed in transcription of genes which products are involved in reproductive processes: homeodomain-like superfamily protein (AT1G01060.4), auxin response factor (AT1630330.2) or bHLH protein (AT4G02590.30). Several genes which products are known to participate in biological regulation were strongly up-regulated, e.g. GIGANTEA protein (AT1G22770.1), F-box/RNI-like superfamily protein (AT1G21410.1), MAPK4 protein (AT1G10210.2) or MYB-like TF (AT5G47390.1). Significant increase in transcription during dehydration in CG rhizomes was observed also in genes which products have catalytic activity, such as haloacid dehalogenase-like hydrolase (AT2G32150.1), pheophytinase (AT5G13800.1), xyloglucanendotransglucosylase/hydrolase 13 (AT5G57540.1), receptor-like protein kinase (AT5G40380.1) and nudix hydrolase homolog 8 (AT5G47240.1) as well as in genes with transporter activity such as transmembrane amino acid transporter (AT3G30390.2) or PIP 1;5 (AT4G23400.1). On the other hand, strong down-regulation was observed in transcription of gene encoding phytosylfokine-alpha receptor 2 (AT5G53890.1) known to be involved in controlling cell expansion [20] or alcohol dehydrogenase 1 (AT1G77120.1). Interestingly, transcription of gene enncoding photosystem II subunit QA (AT4G21280.1) as well as transcription of genes coded for proteins with oxidoreductase activity (APS reductase 1, AT4G04610.1; glutathione peroxidase, AT4G11600.1) was up-regulated. Chen et al. [21] suggest that the PSII-LHCII supercomplexes (photosystem II-light harvesting complex II) and LHCII assemblies play an important role in preventing photo-damages to PSII under drought stress.

### Validation of the results obtained by microarray analysis via real-time PCR (qRT-PCR)

**Fig. 7:**
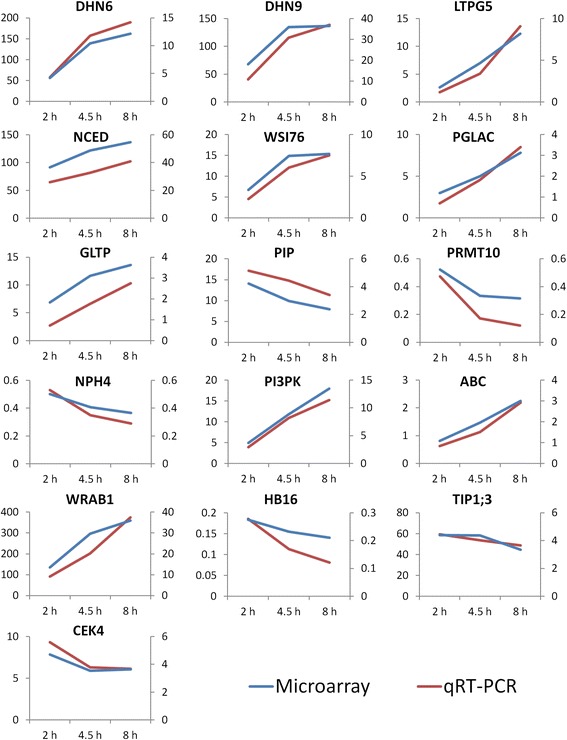
Comparison of qRT-PCR and Microarray results for selected set of genes. Transcription fold changes between treated samples (2, 4.5 and 8 h of dehydration) and non-treated samples (0 h of dehydration) were calculated for both qRT-PCR and Microarray data. qRT-PCR values were obtained by delta Ct method and normalized to selected reference genes. Microarray data were normalized and Log2 fold change values were transformed to non-logarithmic scale for the comparison. Values of transcription fold change bellow 1 depicts the gene down-regulation under particular treatment, while the fold change above 1 shows the up-regulation of gene. X axis depicts the duration of the stress. Primary y axis (left side of the plot) shows the values of transcription fold changes from qRT-PCR (red line in the plot), while secondary y axis (right side of the plot) shows the transcription fold changes as meassured by microarray technique (blue line in the plot). **NPH4** - Transcriptional factor B3 family protein, **PIP** – PIP aquaporin, **PI3PK** - phosphatidylinositol-3P 5-kinase -,**LTPG5** – glycosylphosphatidylinositol-anchored lipid transfer protein 5, **PGLAC** - phospholipid/glycerol acyltransferase, **WSI76** – galactinol synthase, **PRMT10** - histone-arginine-N-methyltransferase, **ABC** - ABC transporter, **NCED** - 9-cis-epoxycarotenoid dioxygenase, **DHN6** – Dehydrin DHN6, **GLTP** – glycolipid transfer protein, **WRAB1** - ABA-inducible protein WRAB1, **DHN9** – dehydrin DHN9, **CEK4 -** choline/ethanolamine kinase 4, **TIP1;3** – tonoplast intrinsic protein 1;3, **HB16** - homeobox protein 16

Sixteen genes stimulated or inhibited by dehydration within couch grass rhizomes were selected for qRT-PCR analysis (Table 3) and results were compared with those obtained from microarray to validate the robustness of our microarray analysis. Genes were selected according to their impact to the main results from the manuscript and those were: ABA biosynthesis gene *NCED (AT1G78390.1)*, strongly up-regulated by dehydration as well as genes induced by ABA such as dehydrins DHN6 (AT4G01985.1), DHN9 (AT1G09350.1), WRAB1 (AT3G15670.1) or galactinol synthase (WSI76; AT1G09350.1), the key enzyme in raffinose biosynthesis. Induced were also genes associated with lipid metabolism such as PI3PK (AT1G34260.1), glycosylphosphatidylinositol-anchored lipid transfer protein 5 (LTPG5; AT3G22600.1), phospholipid/glycerol acyltransferase (PGLAC; AT1G80950.1) and glycolipid transfer protein (GLTP; AT4G39670.1) or genes encoded transporters, e.g. PIP2A (AT3G53420.2), TIP 1;3 (AT4G01470.1) and ABC transporter (ABC; AT1G15520.1). On the other hand, down-regulated were genes coded for NPH4 (AT5G20730.2), HB16 (AT4G40060.1) and PRMT10 (AT1G04870.2). However, CEK4 (AT2G26830.1) was up-regulated as found also by microarray analysis. All the genes gave the same transcription trend as in microarray analysis (Fig. 7 and Additional file 12: Table S1), in some cases even more pronounced – see the very strong up-regulation of dehydrins, WRAB1, TIP 1;3 and NCED as quantified by real time PCR (Fig. 7 and Additional file 12: Table S1).

**Table 3:** Genes stimulated or inhibited by dehydration within couch grass rhizomes selected for qRT-PCR analysis, sequences of the primers and amplification efficiencies

ID^a^	AGI^b^	Name^c^	Primer sequence	Efficiency (%)^d^
Contig15047_at	AT5G20730.2	NPH4_F	atcctatcccctcaagaagtgcaaa	93
		NPH4_R	tggtcgtaacgaggcttccaagtat	
Contig1223_at	AT3G53420.2	PIP_F	agtacgtcctgagggcgagtg	92
		PIP_R	cacgatccgagccatatcacactgat	
Contig14720_s_at	AT1G34260.1	PI3PK_F	gagtttgtacttggcatcatcgact	95
		PI3PK_R	aaccgttaggaaatacttggccatg	
HVSMEf0022D18r2_s_at	AT3G22600.1	LTPG5_F	gatcgggttggcgcgcataca	92
		LTPG5_R	atgcatgtcacggtacaacaaatgga	
Contig10474_at	AT1G80950.1	PLGAC_F	gttgctctttcctgagggcac	108
		PLGAC_R	aaaatgactggttgtactggtgctc	
Contig3810_at	AT1G09350.1	WSI76_F	tacgtgcaagcacacggttgg	97
		WSI76_R	acgtttcagccatgcatacgtgtacg	
Contig14329_at	AT1G04870.2	PRMT10_F	ttgatgactccatctccgagagtaa	94
		PRMT10_R	atccatatccataagccggtgattc	
Contig10182_at	AT1G15520.1	ABC_F	tcagccctattgcatggacactcaa	94
		ABC_R	gctactacccacaggaagtcgtgat	
HT11N18r_s_at	AT1G78390.1	NCED_F	cttattaggcataggagatccccgg	94
		NCED_R	tgaagcaagtgtgagctaactgaat	
Contig1708_s_at	AT4G01985.1	DHN6_F	agcacaagaccggtggcatcct	103
		DHN6_R	tccttgttaccgccggggagct	
HW09B04u_at	AT4G39670.1	GLTP_F	cgttccatagctgggcaatccaga	108
		GLTP_R	acagagcaatcagtttcgttgagcc	
Contig2406_at	AT3G15670.1	WRAB1_F	ttgcctttgatttgatggtactcgtgt	97
		WRAB1_R	gtgccacctttcgactgtcctc	
Contig1718_s_at	AT3G50980.1	DHN9_F	aagacccgtgggatactgcatcgct	96
		DHN9_R	gtcgccatgtgctgctggttgtc	
Contig9547_at	AT2G26830.1	CEK4_F	ggcactcattcaggcaagggta	95
		CEK4_R	ctcctcagtgaagaaaggaagcctt	
HVSMEf0019H18r2_s_at	AT4G01470.1	TIP1;3_F	atccatgcgtcatcgccatga	96
		TIP1;3_R	tgactgactcacacacagtttaccc	
Contig10112_at	AT4G40060.1	HB16_F	gatcctcggacagcgactcgagcg	99
		HB16_R	tgtccaggaacgacgcgccgaa	

^a^Affymetrix 22 K Barley1 GeneChip Genome Array probe set ID

^b^*A. thaliana* locus identifier corresponding to individual IDs

^c^*F* forward, *R* reverse, *NPH4* Transcriptional factor B3 family protein, *PIP* plasma membrane intrinsic protein, aquaporin, *PI3PK* phosphatidylinositol-3P 5-kinase, *LTPG5* glycosylphosphatidylinositol-anchored lipid transfer protein 5, *PGLAC* phospholipid/glycerol acyltransferase, *WSI76* galactinol synthase, *PRMT10* histone-arginine-N-methyltransferase, *ABC* ATP-binding cassette transporter, *NCED* 9-cis-epoxycarotenoid dioxygenase, *DHN6* dehydrin DHN6, *GLTP* glycolipid transfer protein, *WRAB1* ABA-inducible protein WRAB1, *DHN9* dehydrin DHN9, *CEK4* choline/ethanolamine kinase 4, *TIP1;3* tonoplast intrinsic protein 1;3, *HB16* homeobox protein 16

^d^Amplification efficiency

## Discussion

Plenty of studies have been performed to explore stress adaptational mechanisms of cultivated crop species on a genome scale. However, stress responses of weedy relatives were investigated much less in the past, partially because of the limited number of genomic tools for weedy species. We used Barley1 GeneChip genome array to explore stress responses of CG (relative of barley), one of the most problematic weed species. Considering that almost half of the features on Barley1 GeneChip were called present once hybridized with CG RNA confirmed the applicability of barley microarray for CG stress response analysis, despite there is only partial homology of barley and CG genomes [5–7]. In addition, the CG homologs in barley genome involved in stress response was identified. Obtained information could contribute to both crop improvement and better weed management practice [22, 23]. Here we tested the hypothesis that the dehydration stress response of barley crown and the node of its wild extremely tolerant relative (couch grass) is different. We discuss mechanisms of acclimation to dehydration which play a crucial role in couch grass node, plant part critical for whole plant survival, similar to barley crown.

CG rhizomes are well adapted to regenerate following a period of moisture stress. The molecular basis of this adaptation was sought by subjecting rhizome segments to a range of simulated dehydration intensities, and documenting the induced changes to their transcriptome. The strategy was based on similar experiments designed to characterize the response to dehydration stress of the barley crown [16]. The resilience of the CG rhizomes was shown by the fact that, despite their partial dehydration, they retained a substantial degree of viability, according with the observations of Mikulka and Kneiflová [24]. The transcriptomic analysis revealed that the number of reprogrammed genes rose as the intensity of the dehydration was increased. The prominent role of ABA-responsive genes in dehydration stress response was evident in all the intervals of CG stress treatment together with the strong up-regulation of ABA synthesis genes - both *NCED* (AT1G78390.1) and *zeaxanthin epoxidase* (AT5G67030.1) were strong up-regulated and this up-regulation was specific for couch grass in our experiment. Especially the very strong up-regulation of *NCED* might be the important step in CG dehydration response regarding its regulatory role in ABA biosynthesis [25].

Among the ABA-inducible genes up-regulated by the stress were a number encoding LEA proteins. The intensity of transcription of *HVA1* (AT3G15670.1), a barley member of this group of proteins, has been shown to be positively correlated with drought tolerance not just in barley, but also in other plant species [26–31]. *WSI18* (AT3G15670.1) is another *LEA* gene thought to be drought-responsive [32–34], while the LEA14-A (AT1G01470.1) protein possesses a level of sequence identity with a protein induced in the leaf and root of the resurrection plant *Craterostigma plantagineum* during desiccation and following the exogenous supply of ABA, as well as in callus challenged with NaCl [35]. The suggestion is that the stress-induced up-regulation of these (and other) genes contributes to the high level dehydration tolerance shown by CG rhizomes.

Given that a common plant response to dehydration (and certain other abiotic stresses) is to accumulate sugars and other compatible solutes [36], it was not unexpected to find that genes encoding UDP-glucose 4-epimerase (UGE; AT4G23920.1)*,* a putative trehalose-6-phosphate phosphatase (AT4G12430.1), galactinol synthase (AT1G09350.1) and a putative raffinose synthase (AT5G40390.1) were all significantly up-regulated in the CG rhizomes by the simulated dehydration stress. The enzyme UDP-glucose 4-epimerase catalyzes the final step of galactose metabolism. In *A. thaliana*, genes encoding this protein appear to be induced by drought, low temperature and salinity stress [37], and some UGE isoforms have been shown to be involved in the stress response [38]. The rice homlog *OsUGE-1* can be induced by various abiotic stress agents [39]. The implication is therefore that producing a high level of UGE activity in the CG rhizomes can represent an adaptation to dehydration stress. Trehalose-6-phosphate phosphatase (TPP) processes trehalose 6-phosphate to produce trehalose, an important disacharide in the context of acquiring drought tolerance: a positive correlation has been established between the abundance of TPP transcript and drought tolerance in rice [40, 41]. Both galactinol synthase and raffinose synthase are involved in the synthesis of the raffinose oligosacharides, which act as osmoprotectants: their beneficial effect for plants exposed to drought has been well documented [42–44]. Proline is probably the most well studied compatible solute. The over-accumulation of proline by transgenic rice plants over-expressing a gene encoding δ-1-pyrroline-5-carboxylate synthase (AT2G39800.1) was correlated with the plants’ improved capacity to withstand both moisture and salinity stress [45]. Similarly, transgenic tobacco plants constitutively expressing a homolog from *Vigna aconitifolia* produced more proline than wild type plants, resulting in a boost to both root growth and seed yield under conditions of drought stress [46]. The strong up-regulation of this gene in the CG rhizomes is thus consistent with proline accumulation being an aspect of the structure’s adaptation to dehydration.

Genes encoding a number of proteins involved in stress signal transduction were also up-regulated in the CG rhizomes by the simulated dehydration stress treatments; one of these was a homolog of *PKABA1* (AT4G33950.1). In non-stressed wheat seedlings, *PKABA1* is transcribed at a very low level, but this changes dramatically when they are exposed to abiotic stress [47, 48]. Another example, protein phosphatase 2C (AT1G72770.3, AT4G28400.1, AT4G31750.1, AT1G72770.3) is considered to be a negative regulator of ABA: the accumulation of transcripts occurs in response to moisture stress in barley [49, 50]. *PI3PK* (AT1G34260.1) is known to be up-regulated in barley roots exposed to prolonged salinity stress [51], while in *A. thaliana PIP5K* is rapidly induced by exposure to drought, salinity and exogenous ABA [52].

The barley protein Blt 101.1 (AT2G38905.1), strongly up-regulated in CG, belongs to a gene family whose members are responsible for the maintenance of plasma membrane potential during a stress episode [53]. Its encoding gene is strongly transcribed in the vascular-transition zone of the barley crown [54], considered to be the part of the mature plant most sensitive to freezing damage. The protein has been described as freezing-, but not drought-responsive [55], although its response to dehydration in the crown has not been systematically studied as yet.

The up-regulation of genes encoding various transporters observed in the mild dehydration stress treatment suggested that a stimulation of transport activity may represent an early response to the stress. MIPs (major intrinsic proteins) enable a rapid and reversible alteration in water permeability [56]. Their regulation of water movement during a drought episode has been proposed by Chaumont and Tyermon [57], based on their expression at the apoplastic barrier in the root [58]. In a leaf of drought treated plant, MIPs participate in the regulation of stomatal movement [59], while in the root, plasma membrane intrinsic proteins (PIPs), a sub-class of the MIP family, are generally reduced in abundance, thereby helping to minimize water loss [60]. According to Lian et al. [61] PIPs are less abundant in the root of a drought sensitive rice cultivar than in a resistant one. The importance of PIPs for the recovery of *A. thaliana* from water-deficient conditions has been demonstrated by [62]. Both the CG node and the barley crown, while both being derived from the stem, are sub-surface structures. Their epidermis, unlike that of the root, is covered with a water-impervious cuticle and, unlike leaves, are not photosynthetically active. In addition, crowns and nodes are crucial for plant survival [63, 64], which lead us to the presumption that these parts of plant are preferentially protected. Three transporter genes behaved differently in the comparison between the CG rhizome and the barley crown. These encoded a PIP2A (AT3G53420.2), a VIT 1.1 (AT2G01770.1) and TIP1;3 (AT4G01470.1), and were each down-regulated in the barley tissue (both cultivars), but up-regulated in CG in response to dehydration stress. They may therefore represent a CG-specific stress adaptation. PIPs are believed to have dual ion and water permeability [65] and behave as a platform for recruitment of a wide range of transport activities [66]. TIPs are important players in mediation of water transport across tonoplast, which is important for osmotic adjustment during drought stress response [67]. E.g. it was shown, that the *Thellungiella salsuginea* tonoplast aquaporin TsTIP1;2 functions in protection against multiple abiotic stresses [68]. Increase in water absorption requires not only roots elongation, but also high water permeability in the tonoplast enabled by TIPs [69].

A significant group of DTGs was represented by genes encoding lipid transport proteins (LTPs): most of these were up-regulated by the stress, in some cases in both CG and barley. LTPs regulate vesicular trafficking, signal transduction and lipid metabolism [70]. Examples of these genes were *LTPG5* encoding glycosylphosphatidylinositol-anchored lipid protein transfer 5 (AT3G22600.1), involved in the accumulation of suberin and sporopolenin [71]; a gene encoding GLTP (AT4G39670.1); and a gene encoding a voltage-dependent L-type calcium channel subunit (AT5G16550.1). Some LTP-encoding genes associated with development were also strongly up-regulated: two examples were *TED4* (AT3G18280.1), which participates in xylem development [72] and a homolog of an *A. thaliana* gene encoding PGLAC (AT1G80950.1), which is a component of the regulation of growth [73]. LTPs have been implicated in both freezing and drought tolerance [74, 75], and are also important transporters of lipids to the cuticle [69]. Some of the material targeted to the cuticle is transported within oleosomes, structures which are coated by oleosin-like proteins [76]. Their transport through the hydrophilic cell wall is facilitated by LTPs [77, 78]. A gene encoding oleosin (AT4G25140.1) was up-regulated in both the crown of barley cv. Tadmor and the CG rhizome subjected to dehydration stress. Unexpectedly, a gene encoding ABCG11 (AT1G17840.1), a protein required for cutin transport to the extracellular matrix [79], was down-regulated in mildly stressed CG rhizomes, although this was reversed in the stronger stress treatments. Two other genes encoding ABC transporters (AT5G64940.1; AT4G04770.1) were up-regulated in all of the treatments: one of these (AT5G64940.1) is involved not only in lipid synthesis [80], but also in cross talk between ABA and reactive oxygen species signaling [81]. A further five ABC transporter genes were up-regulated by the severe dehydration stress treatment (AT5G60740.1, AT4G39850.2, AT5G60740.1, AT4G39850.1, AT1G15520.1).

Under the most severe level of dehydration stress tested, the transcription of several genes encoding products associated with development and translation were down-regulated, while genes encoding products involved in the synthesis of phenolic compounds behaved in the opposite manner. As shown by the ability of CG rhizomes to retain much of their viability even after being exposed to severe moisture stress, suggesting this processes are still reversible. The more drought tolerant barley cultivar shared some similarities with CG, implying that their response was to remain in the vegetative state and to inhibit root branching as will be explained in the following sentences. The down-regulation of *PRMT10* (AT1G04870.2) and *NPH4* (AT5G20730.2) is consistent with this conclusion and transcription of both these genes was verified by real-time PCR. A similar inhibition of root branching occurs in *A. thaliana* in response to ABA treatment, a partial surrogate for drought stress. [82] have shown that the drought tolerance of maize can be improved by reducing lateral root branching. Harris [83] have demonstrated how ABA is involved in the regulation of root architecture in plants exposed to various stress agents. The up-regulation of a gene encoding a phosphatidyl serine synthase in CG was not mirrored in barley in our experiment. The translocation of phosphatidyl serine between organelles and its exposure to the cytosol have been suggested to be important for development [84]. Note that a gene encoding PI3PK (AT1G34260.1) was also strongly up-regulated in dehydration-treated CG rhizomes. Hirano et al. [19] have reported that in *A. thaliana,* mutations in this gene lead to an impairment of endomembrane homeostasis, resulting in a number of pleiotropic developmental abnormalities. Rapid ABA-induced stomatal closure requires the presence of phosphatidylinositol 3,5-bisphosphate, a product of phosphatidylinositol-3P 5-kinase [85]. A further strongly up-regulated gene in CG encoded 1-deoxy-D-xylulose 5-phosphate reductoisomerase (AT5G62790.1), an enzyme participating in isoprenoid synthesis; the isoprenoids represent a diverse group of metabolites involved in photosynthesis, the regulation of growth and interactions with the environment [86]. The product of a gene encoding CEK4 (AT2G26830.1), which was up-regulated in CG but not in barley, catalyzes the initial steps of phospholipid synthesis [87].

## Conclusion

Given that nearly half of the barley probe sets were informative for couch grass, accessing the transcriptome of a crop wild relative using a commercially available chip would appear to represent a simple means of identifying the set of genes associated with a particular process, such as in this case the dehydration tolerance of couch grass. Such information could be of interest not only in the context of improving the performance of a crop species, but also of devising strategies aimed at controlling the growth of weeds.

The results of this transcriptomic survey are consistent with ABA having a prominent role in the drought stress response, along with exerting an influence over development and lipid metabolism. Our results pointed out the important role of transporters and re-programming of the developmental process and root architecture mediated by ABA. The analyzed meristematic part (crown and node) is the main part of plant that had to survive in case of the whole plant survival. Its survival is probably (according to transcriptomic data) connected not only with altered root architecture suggesting altered water output, but also with consolidation of the lipidic barrier on the node surface and altered lipidic metabolism as well as altered activity of water channels and other transporters. These adaptations are according to transcriptomic data suggested to be greatly developed in the meristematic nodes of couch grass. The main results were confirmed via real-time PCR.

## Methods

### Plant material and drought treatments

CG rhizomes were collected from the field (trials were carried out in Prague-Ruzyně - local soil type Orthic Luvisol; average conditions of the field from the day of collection: 14.1 °C; wind 8.3 m/s; rainfall 1.2 mm), washed and cut into 2 cm segments, centered on a single node (the meristematic section of the segment). Non-treated segments were snap-frozen in liquid nitrogen and stored at − 80 °C, and the remaining were subjected to a range of dehydration treatments, comprising exposure to either 2 h, 4.5 h, 8 h and 22 h at 28 °C, chosen to simulate an episode of, respectively, mild, medium, severe and lethal dehydration. At the end of the treatment, the node was subjected to a series of physiological assays (see below), while the rest of the segment was snap-frozen and stored at − 80 °C. The frozen material was used as a source of RNA, extracted using the TRIzol reagent (Invitrogen, Carlsbad, CA, USA), and purified using an RNeasy column in the presence of DNase (Qiagen, Hilden, Germany). The RNA’s quality was assessed through both agarose gel electrophoresis and the use of an Agilent 2100 Bioanalyzer (Agilent Technologies, Santa Clara, CA, USA). RNA was extracted from three rhizome segments per treatment and each sample was represented by three independent replicates.

### Transcriptomic analysis

The RNA samples were hybridized to an Affymetrix 22 K Barley1 GeneChip Genome Array [88]. Barley1 GeneChip Genome Array is designed as a matrix of 712*712 features (506944). There is 502,874 oligonucleotide probes located on the chip. Each probe has 25 bases in length. Sequence of each probe is based on the consensus sequence of selected barley genes. Probe sets corresponding to individual genes are represented by 11 probes 100% complementary to reference sequence (PM; perfect match probes) and by 11 probes complementary to reference sequence except for the middle base (MM; mismatch probes). Only probe sets serving as a control are represented by 20 PM and 20 MM probes. The array contains probes corresponding to 22,840 genes.

Standard controls provided by the supplier were included and B2 oligonucleotides were added to each hybridization cocktail. PolyA controls (lys, phe, thr, dap) and hybridization controls (BioB, BioC, BioD and Cre) were used to monitor labeling and hybridization. Open-source software included within the R statistical package [89] were used for the statistical analysis of the chip outputs. The microarray raw data were subjected to pre-processing analysis using functions provided with the Affy package library [90], with emphasis on boxplots, density plots and Bland-Altman plot modification (MVA plot). Subsequently, the RMA method [91] was implemented to achieve normalization and to eliminate background noise and processing artefacts. An iterative median polishing procedure was used to summarize the data and to generate a single expression value for each probe set. The MAS 5.0 algorithm within the R “Affy” library was used to associate a “present”, “marginal” or “absent” call for each probe, and this output was used as an initial filter to ensure that only calls for which all three replicates were recorded as present in at least one sample were retained. Differentially transcribed genes (DTGs) were defined as those for which the difference in transcript abundance was at least two fold; these were identified using a linear model for microarray analysis provided within the LIMMA library package [92]. Pairwise analyses of treated vs. parallel non-treated samples were accomplished, applying a *P* value threshold of 0.05. A principal component analysis (PCA) was carried out using routines implemented in the Amap library package [93]. DTGs which were either specific to or shared between treatment were visualized using a Venn diagram [94]. The same tool enabled the identification of specificity/commonality between CG and barley. Clusters of genes sharing similar transcriptional patterns were identified for a set of DTGs common to all of the dehydrated CG rhizome segments. Over-represented functional gene categories within each cluster were determined using GO enrichment analysis (http://bioinfo.cau.edu.cn/agriGO/). DTG annotation was achieved using the Plexdb tool (www.plexdb.org)**.** Consensus sequences of selected probes were subjected to BLAST search against Uniref90 (www.uniprot.org) using BlastX, applying an e value threshold of 1e-4. *A. thaliana* identifiers (AGIs) (corresponding to Affymetrix identification numbers) of all of the DTGs were acquired from www.harvest.ucr.edu.

### Two-step real-time reverse transcription PCR (qRT-PCR)

RNA was diluted to 150 ng μl^− 1^ of which a 2 μl aliquot was used as template in a reverse transcription reaction carried out in a volume of 100 μl using TaqMan Reverse Transcription Reagents (Applied Biosystems, Foster City, CA, USA), according to manufacturer’s protocol. A 2 μl aliquot of the reaction product was then taken as the template for a subsequent 20 μl qRT-PCR containing 7.2 μlH2O, 10 μl Power SYBR Green PCR Master Mix (Applied Biosystems, USA) and 200 nM of each relevant primer. The amplification regime comprised a 10 min denaturation at 95 °C, followed by 40 cycles of 95 °C/15 s and 60 °C/60 s. The signal was recorded during the annealing phase of each cycle. Melting curves of PCR products were also recorded. The specificity of the amplicon was checked by electrophoresis through a 2% *w*/*v* agarose gel and the melting curves were evaluated (data not shown). Three technical replicates of each biological sample (3 biological replicates of both treated and non-treated samples) were included.

We designed primer sequences for six candidate reference genes (RGs) used successfully in barley stress response studies before (Table 4). The suitability of each candidate reference gene was evaluated using three programs implemented within Microsoft Excel, namely GeNorm [95], NormFinder [96] and BestKeeper [97]. Because the ranking of the RGs tested differed depending on which algorithm was used (see Additional file 13: Table S2); thus, we also used RefFinder (http://leonxie.esy.es/RefFinder/?type=reference#), a web-based tool that integrates the outcomes of four different algorithms, namely GeNorm, NormFinder, BestKeeper and comparative Delta Ct method [98]. The genes are ranked according to the individual algorithms in RefFinder, and the geometric mean is calculated based on a particular weighting. The genes with the lowest value are considered the most stable. The final ranking of candidate reference genes according to GeNorm, NormFinder and BestKeeper as well as RefFinder is displayed in Additional file 13: Table S2.

**Table 4:** Genes from which the primer sequences of candidate reference genes were derived, sequences of the primers and amplification efficiencies

ID^a^	AGI^b^	Name^c^	Primer sequence	Efficiency (%)^d^
Contig1390_3_s_at	AT5G09810.1	ACT_F	tcactcagcactttccaacagatgt	105
		ACT_R	gactagatgataacagcagtggagc	
Contig2580_3_s_at	AT1G69410.1	IF5A_F	tttgggacccttgtgtttcctatgg	95
		IF5A_R	tttctggcatacagtttgcaccgtc	
Contig1008_x_at	AT5G14670.1	ADP-RF_F	tagttctctcgggatgtcgggggtc	91
		ADP-RF_R	gacaaaaatgagaccctgggtgttctga	
Contig21863_at		HSP90_R	ggacgctgtttattggctacgacga	103
		HSP90_F	tccatacacacagtcgggacgtatc	
Contig306_s_at	AT5G45350.2	SIGPRP_F	taccctggctcatctggtcacagtg	90
		SIGPRP_R	agagatcttgtgtgctccgtaagcg	
Contig149_at	AT1G13440.1	GAPDH_F	gggttcccactgtggatgtgtcagt	91
		GAPDH_R	ttccctcggaagcagccttgatagc	

^a^Affymetrix 22 K Barley1 GeneChip Genome Array probe set ID

^b^*A. thaliana* locus identifier corresponding to individual IDs

^c^*F* forward, *R* reverse, *ACT* Actin, *IF5A* Translation elongation factor 5A, *ADP-RF* ADP-ribosylation factor, *HSP90* cytosolic heat shock protein 90, *SIGPRP* similar to *GPRP* (proteins rich in glycine and proline), *GAPDH* glyceraldehyde-3-phosphate dehydrogenase

^d^Amplification efficiency

To find out the optimal number of reference genes, we applied the pairwise variation (V) within GeNorm (see Additional file 14: Figure S1). The pairwise variation *V* is based on the comparison between NFn (normalization factor) of the most stable control genes and NF_n + 1_, reflecting the effect of additional gene (n + 1) inclusion. The inclusion of additional RGs is recommended when the variation exceeds the 0.15 cut-off value. If the variation is below this limit, the inclusion of another gene is not required [95]. Therefore, we choose combination of two most stable reference genes (GAPDH, ADP-RF) suitable enough for normalization, since the V2/3 value (0.100) was below the limit for additional gene inclusion.

The qRT-PCR efficiency for each target gene was calculated using qRT-PCR 10-fold serially diluted cDNA in triplicate and the following formula: *E = (10*^–1/S^*100)-100 where *E* is amplicon efficiency and *S* is a slope of standard curve. Only sequences associated with the efficiency of > 90% were taken forward (Table 4).

Several genes stimulated/inhibited by dehydration within couch grass rhizomes were selected for qRT-PCR analysis (Table 3). Results were compared with those obtained from microarray to validate the data. Target cDNA sequences were derived from contig probe sequences within Affymetrix Barley1 GeneChip Genome Array. PCR primers were designed using FastPCR software (Primer design Ltd., Finland), and their specificity was verified by a BLAST search of the NetAffx™ Analysis Center and NCBI databases.

### Calculation of normalized transcription of selected genes in qRT-PCR and comparison between qRT-PCR and microarray data

Real time transcription values was calculated using delta Ct method. Relative transcript abundance was calculated using the formula: $\mathit{\mathsf{Q}}={\mathit{\mathsf{E}}}^{\left({\overline{Ct}}_{0h}-{\overline{Ct}}_{sample}\right)}$ where $\mathit{\mathsf{Q}}$ is a relative transcript abundance, $\mathit{\mathsf{E}}$is amplification efficiency of gene of interest (GOI), ${\overline{\mathit{\mathsf{Ct}}}}_{0h}$is a Cycle threshold average value in non-treated samples (0 h of dehydration) and ${\overline{\mathit{\mathsf{Ct}}}}_{sample}$ is cycle threshold average value in the specific treated sample. In the next step, the values of normalized transcription (${\mathit{\mathsf{CG}}}_{NORM}$) was generated using the formula: ${\mathit{\mathsf{CG}}}_{NORM}=\frac{{\mathit{\mathsf{Q}}}_{CG}}{NF_N}$ where $\mathit{\mathsf{Q}}$ is a relative transcript abundance of GOI in specific treated sample and $\mathit{\mathsf{NF}}$ is a normalization factor ${\mathit{\mathsf{N}\mathsf{F}}}_{\mathit{\mathsf{N}}}=\frac{1}{\mathit{\mathsf{N}}}\sum \limits_{i=1}^N{\mathit{\mathsf{Q}}}_{REF}$, representing the geometric mean of relative transcript abundances of selected reference genes (${\mathit{\mathsf{Q}}}_{REF}$) genes in individual treated samples. $\mathit{\mathsf{N}}$ is a number of reference genes.

The differences in the gene transcription between treated and non-treated samples on the microarray generated by the LIMMA algorithm are Log2 transformed. For their comparison with the real time the fold changes were transformed to non-logarithmic scale using the formula ${2}^{\log 2\mathit{\mathsf{FC}}}$.

### Physiological analyses

The rhizome material surrounding the node was used to acquire key physiological parameters. These were: relative water content (RWC), calculated from the sample’s fresh weight (FW) and dry weight (DW), according to the formula 100 x (FW-DW)/DW; electrolyte leakage, using a modified version of the protocol developed by Prášil and Zámečník [99], which produced the index I_t_ derived from the formula 100 x (R_t_-R_0_)/(R_f_-R_0_), where R_0_ was the electric conductivity of the sample before treatment, R_f_ the conductivity of the sample after 22 h of dehydration treatment (lethal) and R_t_ the relative conductivity expressed by the formula100 x L_t_/L_tm,_ where L_t_ is the amount of electrolyte leakage from a treated sample and L_tm_ is the maximal (total) leakage from treated sample (after killing the same sample by boiling). The degree of damage inflicted by the treatment on the rhizome was further quantified by examining the proportion of replanted treated rhizome segments to sprout when replanted in well-watered soil over a period of 7, 14, 21 and 28 days.

## Additional files

Additional file 1: **Table S3.** List of all genes within cluster 1. Average log2 transcriptions of individual probe sets under individual treatments are displayed as well as log2 FC of individual treatments against non-treated samples. Manufacturer annotation of individual IDs along with HarvEST annotation of individual AGIs is included. (XLSX 41 kb)

Additional file 2: **Table S4.** List of all genes within cluster 2. Average log2 transcriptions of individual probe sets under individual treatments are displayed as well as log2 FC of individual treatments against non-treated samples. Manufacturer annotation of individual IDs along with HarvEST annotation of individual AGIs is included. (XLSX 23 kb)

Additional file 3: **Table S5.** List of all genes within cluster 3. Average log2 transcriptions of individual probe sets under individual treatments are displayed as well as log2 FC of individual treatments against non-treated samples. Manufacturer annotation of individual IDs along with HarvEST annotation of individual AGIs is included. (XLSX 22 kb)

Additional file 4: **Table S6.** List of all genes within cluster 4. Average log2 transcriptions of individual probe sets under individual treatments are displayed as well as log2 FC of individual treatments against non-treated samples. Manufacturer annotation of individual IDs along with HarvEST annotation of individual AGIs is included. (XLSX 13 kb)

Additional file 5: **Table S7.** List of all genes within cluster 5. Average log2 transcriptions of individual probe sets under individual treatments are displayed as well as log2 FC of individual treatments against non-treated samples. Manufacturer annotation of individual IDs along with HarvEST annotation of individual AGIs is included. (XLSX 15 kb)

Additional file 6: **Table S8.** List of all genes within cluster 6. Average log2 transcriptions of individual probe sets under individual treatments are displayed as well as log2 FC of individual treatments against non-treated samples. Manufacturer annotation of individual IDs along with HarvEST annotation of individual AGIs is included. (XLSX 12 kb)

Additional file 7: **Table S9.** List of all genes within cluster 7. Average log2 transcriptions of individual probe sets under individual treatments are displayed as well as log2 FC of individual treatments against non-treated samples. Manufacturer annotation of individual IDs along with HarvEST annotation of individual AGIs is included. (XLSX 9 kb)

Additional file 8: **Table S10.** List of all genes within cluster 8. Average log2 transcriptions of individual probe sets under individual treatments are displayed as well as log2 FC of individual treatments against non-treated samples. Manufacturer annotation of individual IDs along with HarvEST annotation of individual AGIs is included. (XLSX 13 kb)

Additional file 9: **Table S11.** List of all genes within cluster 9. Average log2 transcriptions of individual probe sets under individual treatments are displayed as well as log2 FC of individual treatments against non-treated samples. Manufacturer annotation of individual IDs along with HarvEST annotation of individual AGIs is included. (XLSX 10 kb)

Additional file 10 **Table S12.** List of all genes within cluster 10. Average log2 transcriptions of individual probe sets under individual treatments are displayed as well as log2 FC of individual treatments against non-treated samples. Manufacturer annotation of individual IDs along with HarvEST annotation of individual AGIs is included. (XLSX 9 kb)

Additional file 11: **Table S13.** List of all DTGs (810) specific to Couch Grass treated samples. Log2 FC of individual treatments against non-treated samples are displayed along with. Manufacturer annotation of individual IDs and HarvEST annotation of individual AGIs. (XLSX 122 kb)

Additional file 12: **Table S1.** Comparison of qRT-PCR and Microarray results for selected set of genes. Comparison of qRT-PCR and Microarray results for selected set of genes. Transcription fold changes between treated samples (2, 4.5 and 8 h of dehydration) and non-treated samples (0 h of dehydration) were calculated for both qRT-PCR and Microarray data. qRT-PCR values were obtained by delta Ct method and normalized to selected reference genes. Microarray data were normalized and Log2 fold change values were transformed to non-logarithmic scale for the comparison. Values of transcription fold change bellow 1 depicts the gene down-regulation under particular treatment, while the fold changes above 1 shows the up-regulation of gene under particular treatment. NPH4 - Transcriptional factor B3 family protein, PIP – PIP aquaporin, PI3PK - phosphatidylinositol-3P 5-kinase -,LTPG5 – glycosylphosphatidylinositol-anchored lipid transfer protein 5, PGLAC - phospholipid/glycerol acyltransferase, WSI76 – galactinol synthase, PRMT10 - histone-arginine-N-methyltransferase, ABC - ABC transporter, NCED - 9-cis-epoxycarotenoid dioxygenase, DHN6 – Dehydrin DHN6, GLTP – glycolipid transfer protein, WRAB1 - ABA-inducible protein WRAB1, DHN9 – dehydrin DHN9, CEK4 - choline/ethanolamine kinase 4, TIP1;3 – tonoplast intrinsic protein 1;3, HB16 - homeobox protein 16. (XLSX 12 kb)

Additional file 13: **Table S2.** Ranking of candidate reference genes according to used algorithms (GeNorm, NormFinder, BestKeeper and RefFinder). (XLSX 9 kb)

Additional file 14: **Figure S1.** Determination of the optimal number of reference genes for normalization using GeNorm Pairwise variation. The inclusion of additional RGs is recommended when the variation exceeds the 0.15 cut-off value, reppresented by the blue line within the plot. Since this is not the case and the value for V2/3 is bellow the limit, combination of two most stable reference genes was used for the normalization of GOI. (JPG 160 kb)

## Abbreviations

ABA: Abscisic acid
ABC transporter: ATP-binding cassette transporter
CEK4: Choline/ethanolamine kinase 4
CG: Couch grass
CG_norm_: values of normalized transcription
DHN: Dehydrin
DM: Dry matter
DTGs: Differentially transcribed genes
DW: Dry weight
FW: Fresh weight
GLTP: Glycolipid transfer protein
HB16: Homeobox protein 16
LEA: Late embryogenesis abundant proteins
LHCII: Light-harvesting complex II
LTPG5: Glycosylphosphatidylinositol-anchored lipid transfer protein 5
LTPs: Lipid transport proteins
NCED: 9-cis-epoxycarotenoid dioxygenase
NPH4: Transcriptional factor B3 family protein
PCA: Principal component analysis
PGLAC: Phospholipid/glycerol acyltransferase
PI3PK: Phosphatidylinositol-3P 5-kinase
PIP: Plasma membrane intrinsic protein, aquaporin
PRMT10: Histone-arginine-N-methyltransferase
PSII: Photosystem II
qRT-PCR: real-time PCR
RWC: Relative water content
TIP: Tonoplast intrinsic protein
VIT: Vacuolar iron transporter
WRAB1: ABA-inducible protein WRAB1
WSI76: Galactinol synthase
