# Supplementary figures and images for: The dehydration stress of couch grass is associated with its lipid metabolism, the induction of transporters and the re-programming of development coordinated by ABA
Source: BMC Genomics. 2018 May 2;19:317. doi: 10.1186/s12864-018-4700-3 (PMC5930771; doi:10.1186/s12864-018-4700-3)

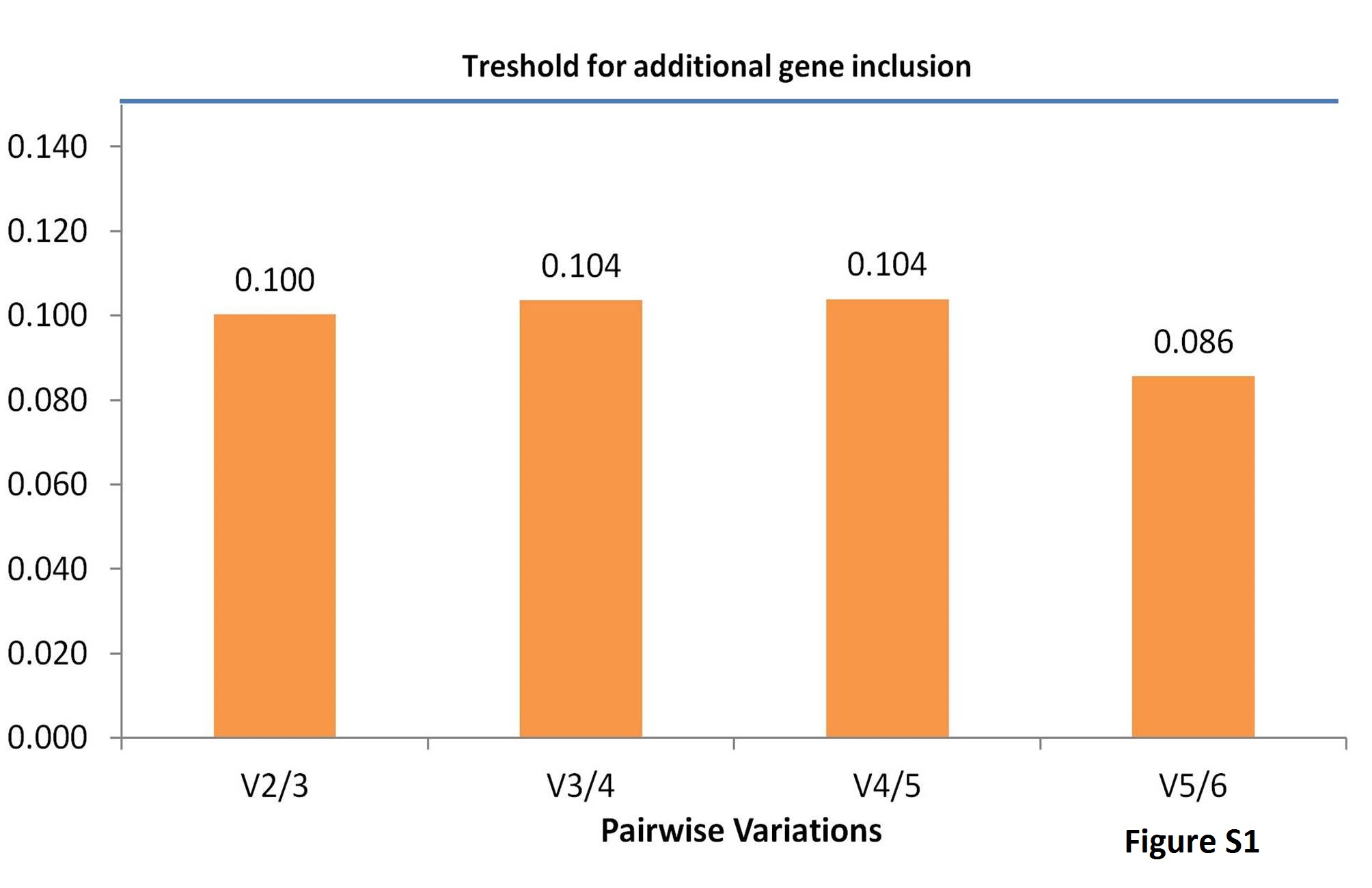

Supplement: Supplementary file 14 — Figure S1. Determination of the optimal number of reference genes for normalization using GeNorm Pairwise variation. The inclusion of additional RGs is recommended when the variation exceeds the 0.15 cut-off value, reppresented by the blue line within the plot. Since this is not the case and the value for V2/3 is bellow the limit, combination of two most stable reference genes was used for the normalization of GOI. (JPG 160 kb) [file 12864_2018_4700_MOESM14_ESM.jpg]
